# The Postage of Periodicals

**Published:** 1899-02-04

**Authors:** 


### THE POSTAGE OF PERIODICALS.

It may be hoped that the new Secretary to the Post Office enters upon his career at St. Martin's-le-Grand with his hands unencumbered by the unyielding fetters of red tape. In spite of all Mr. Henniker Heaton's efforts, the department has by no means fully learnt the lesson that the Post Office exists for the benefit of the British nation, not the British nation for the purpose of paying the salaries of the Post Office staff. But it has made a little progress in that direction; and, with constant spurring, the pace may be considerably forced. There is no doubt that it wants forcing just now in reference to the Postage of Periodicals. The committee of the London Chamber of Commerce, who are engaged in studying the question, and the consequences of the present unequal regulations on trade, will probably contribute to that result, especially if they are instrumental in bringing the matter before Parliament; for the inequality of the regulations only wants, we believe, to be thoroughly grasped in order to be remedied. It can be made clear in a very few words. At present, while any publication which answers to the description of a newspaper, however bulky it may be, is carried for a halfpenny, any publication which is carried under the designation of a periodical is charged at book-rates. Thus, the *Queen* gets through for a halfpenny, and the *Bazaar* must bear a stamp for three-halfpence. Of course, it is right that the *Queen* should get through for a halfpenny, but it cannot be right that the subscribers to the *Bazaar* should be compelled to pay three-halfpence. The only plausible defence for this glaring anomaly is that no halfpenny business pays. But it is too late in the day to enter this plea, even if Mr. Henniker Heaton had not conclusively proved that it is the fault of the Post Office if it does not pay. There is indirect, as well as direct, payment, and periodicals containing a large number of advertisements must, in the nature of things, be the means of bringing grist to the Post Office mill. To attempt to limit their circulation by imposing prohibitive rates of postage is not only reactionary, but short-sighted policy. It should be the policy of the Post Office to encourage by every means in its power the circulation of every publication which is likely to promote the expansion of home industries, instead of persisting in regulations which cannot fail to have the effect of crippling them.

